# Correction: GPR18 Agonist Resolvin D2 Reduces Early Brain Injury in a Rat Model of Subarachnoid Hemorrhage by Multiple Protective Mechanisms

**DOI:** 10.1007/s10571-024-01475-4

**Published:** 2024-04-24

**Authors:** Tongyu Zhang, Gang Zuo, Hongqi Zhang

**Affiliations:** 1 Department of Neurosurgery, Xuanwu Hospital, Capital Medical University, 45 Changchun St., Beijing, 100053 China; 2 Department of Neurosurgery, The Affiliated Taicang Hospital, Soochow University, Taicang, Suzhou, 215400 Jiangsu China

**Correction: Cellular and Molecular Neurobiology (2022) 42:2379–2392** 10.1007/s10571-021-01114-2

The original version of this article unfortunately contained error in Fig. 8 as the two DAPI images in Fig. 8b should be reversed.

The corrected figure is presented here.

**Fig. 8:**
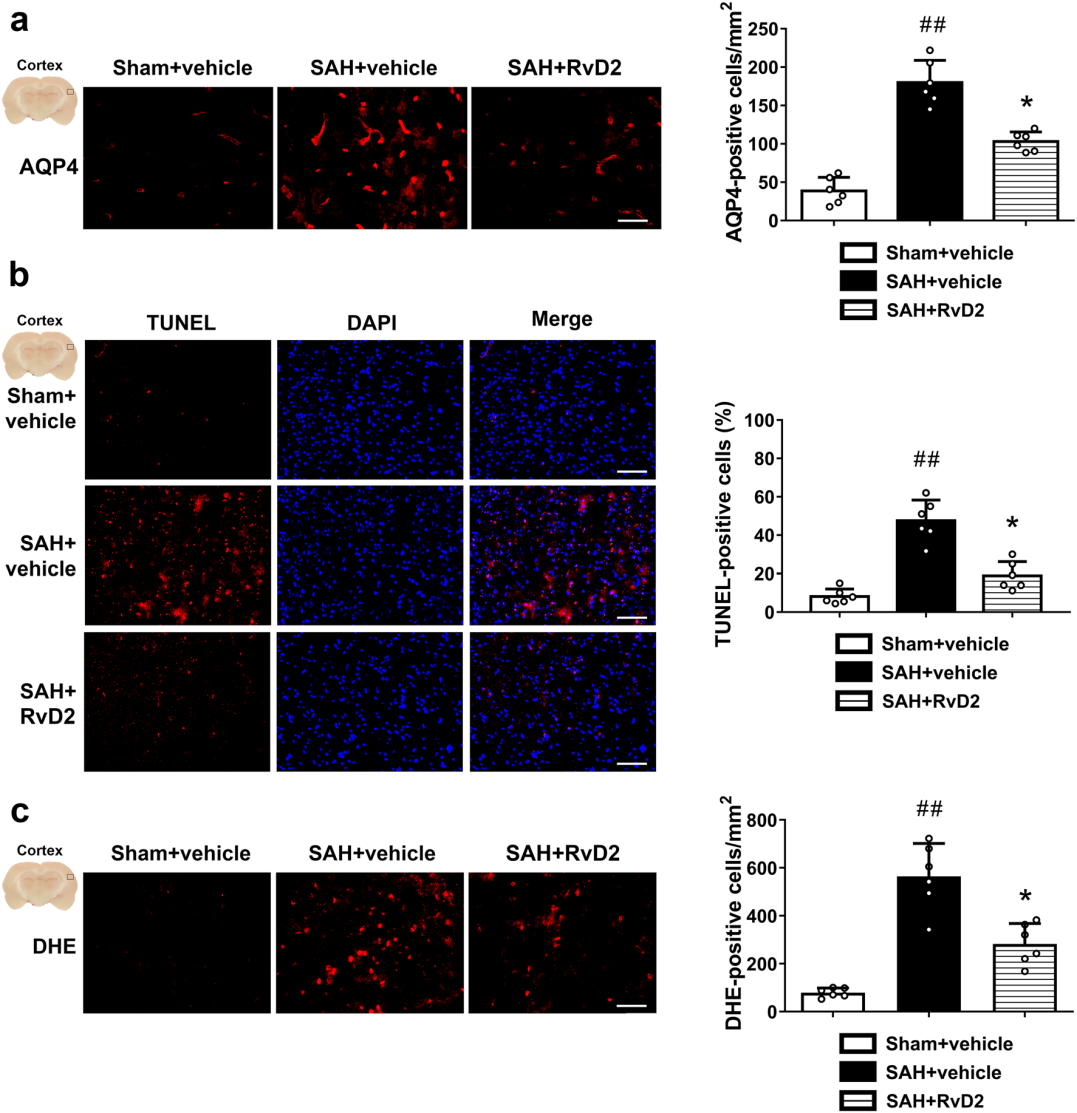
Immunostaining studies to show that RvD2 protected BBB and attenuated apoptosis and oxidative stress in cortex after SAH. **a** Representative immunostaining for AQP4, and quantitative analysis of AQP4-positive cells. **b** Representative TUNEL staining, and quantitative analysis of apoptotic index (percentage of TUNEL-positive cells). **c** Representative DHE staining, and quantitative analysis. Scale bars = 200 μm. Bars represent the mean ± SD (n = 6 from each group). ^##^*P* < 0.01 vs. Sham + vehicle group; *P < 0.05 vs. SAH + vehicle group

The original article has been corrected.

